# Acute pulmonary embolism: a paradigm shift in interventional treatment and interdisciplinary care?

**DOI:** 10.1007/s00330-025-11548-7

**Published:** 2025-05-09

**Authors:** Willie M. Lüdemann, Federico Collettini, Uli Fehrenbach, Timo A. Auer, Maximilian de Bucourt, Bernhard Gebauer

**Affiliations:** Department of Diagnostic and Interventional Radiology, Charité Universitätsmedizin Berlin, corporate member of Freie Universität Berlin and Humboldt-Universität zu Berlin, Augustenburger Platz 1, Berlin, Germany

**Keywords:** Pulmonary embolism, Right heart failure, Shock, Thrombectomy

## Abstract

**Abstract:**

Catheter-based recanalization procedures have long been standard of care in treating myocardial infarction and stroke. Interventional treatments for pulmonary embolism (PE), however, have only been performed occasionally as second-line strategies until recently. Current guidelines still recommend systemic thrombolysis, which may halve the mortality risk in patients with high-risk pulmonary embolism but is underused outside experienced centers. Novel devices for thrombectomy have significantly changed clinical practice and potentially fill a treatment gap in intermediate and high-risk pulmonary embolism. Observational data are encouraging and randomized controlled trials are underway to prove safety and efficacy, which requires the adaptation of current treatment paradigms. Interdisciplinary pulmonary embolism response teams are needed to provide individualized care, and interventional radiologists are suited to play a pivotal role given their expertise in both diagnostic imaging and invasive procedures. To live up to this challenge, interventional radiologists must familiarize themselves with both the pathophysiology of acute PE as well as with the principles of care provided by other team members.

**Key Points:**

***Question*** *The recommended treatment of high-risk pulmonary embolism is systemic thrombolysis. New devices for percutaneous aspiration thrombectomy aim to change treatment paradigms and need critical review.*

***Findings*** *Observational data suggests both safety and efficacy of novel thrombectomy devices, randomized controlled trials are underway. The added value of catheter-directed thrombolysis is unclear.*

***Clinical relevance*** *Systemic thrombolysis reduces mortality in patients with high-risk pulmonary embolism but is disproportionately rarely used outside of experienced centers. Percutaneous large-bore pulmonary thrombectomy holds great potential for filling a treatment gap in intermediate and high-risk pulmonary embolism and changing guidelines.*

## Introduction

Acute pulmonary embolism (PE) is the third most common cause of cardiovascular death after myocardial infarction and stroke, and the leading cause of preventable death in hospitalized patients. Systemic thrombolysis is an effective treatment for PE with a high risk of mortality but carries a significant risk for intracranial bleeding and is underused outside experienced centers [1–4]. Novel thrombectomy devices offer new treatment options for patients with elevated risk of mortality. We review the available evidence on large-bore thrombectomy and its potential role in the treatment landscape of acute PE.

## Epidemiology

According to a German registry study from 2020, the incidence of PE increased from 85.3 per 100,000 in 2005 to 108.7 in 2015 [4]. Simultaneously, the in-hospital mortality rate decreased from 20.4 to 13.9%, while the percentage of hemodynamically unstable patients remained stable at about 9%. Between 2005 and 2015, about 11% of hemodynamically stable patients died during the same hospital stay, compared to 78% in the high-risk group. Risk factors include immobilization following trauma surgery, as well as cardiovascular disease. Beyond the acute mortality rate, PE causes significant long-term morbidity. About half of the patients experience post-PE syndrome, which refers to symptoms like chest pain, shortness of breath, fatigue, dizziness, or fainting for more than 3 months after an acute PE [5]. Approximately 3–4% of these patients develop chronic thromboembolic pulmonary hypertension (CTEPH), characterized by a mean pulmonary arterial pressure greater than 25 mmHg with a pulmonary artery occlusion pressure of 15 mmHg or less, along with ventilation-perfusion defects and confirmed thromboembolic changes in imaging after 3 months of effective anticoagulation [6].

## Pathophysiology of life-threatening pulmonary artery embolism

**Fig. 1:**
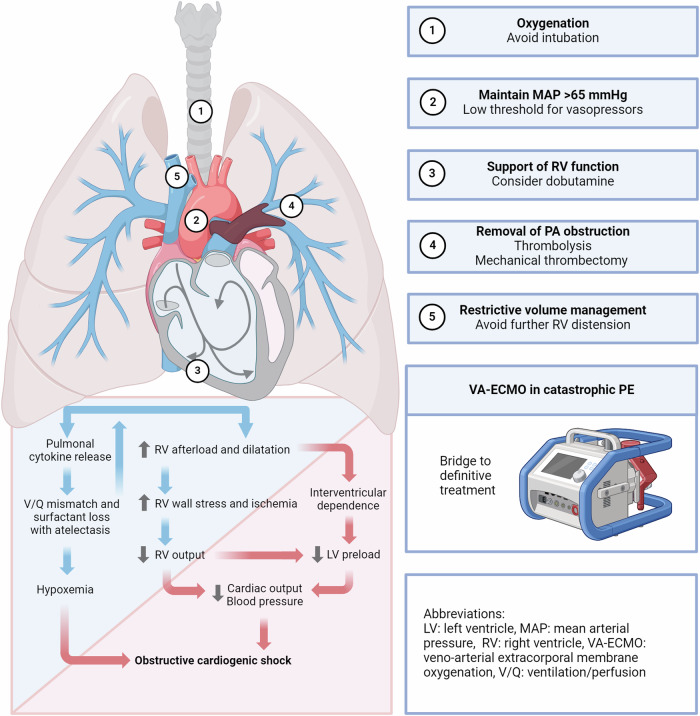
Pathophysiology and management strategies in elevated-risk pulmonary embolism. Created in BioRender. Lüdemann, W. (2025) https://BioRender.com/v99x345

During acute PE, a thrombus typically originating in leg or pelvic veins enters the pulmonary circulation. A sudden increase in right ventricular afterload occurs when pulmonary artery obstruction exceeds 30–50%, which is exacerbated by neurohormonally mediated vasoconstriction [7, 8]. This leads to dilation of the thin-walled right ventricle, increased wall tension and oxygen demand due to myocardial inflammation. The strain-induced malperfusion and relative hypoxia reduce contractility and right ventricular output. Ballooning of the right ventricle shifts the septum, impairing left ventricular filling, lowering output, and reducing systemic blood pressure, further decreasing coronary perfusion. This exacerbates right ventricular oxygen deprivation, potentially leading to obstructive cardiogenic shock due to right heart failure [9] (Fig. 1).

## Risk stratification

Risk stratification is performed using clinical scores, laboratory values and imaging. The pulmonary embolism rule out criteria (PERC) help in cases with a low pre-test probability ≤ 15% while the revised Geneva Score objectifies the risk for PE and helps justify imaging studies. The simplified pulmonary embolism severity index (sPESI) predicts 30-day mortality in PE patients [10–12]. In practice, the revised Geneva Score is most helpful as it is designed to avoid or potentially trigger CT imaging for confirmative diagnosis and definitive risk stratification. The 2019 European Society of Cardiology (ESC) guidelines categorize PE into low, intermediate-low and intermediate-high as well as high estimated acute mortality risk from right heart failure (Table 1) [9]. According to this classification, defining clinical features of at least intermediate-risk PE include a heart rate greater than 110/min, a systolic blood pressure under 100 mmHg, or an oxygen saturation below 90%. The intermediate-high-risk group additionally exhibits elevated cardiac biomarkers and signs of right ventricular dysfunction in echocardiography or CT angiography. Intermediate-low-risk patients meet at most one of these criteria. High-risk patients are characterized by hemodynamic instability, often requiring circulatory support. Treating elevated-risk PE is challenging because patients may decompensate suddenly after prolonged periods of stability.

**Table 1:** Risk stratification of pulmonary embolism

Early mortality risk		Parameter			
		Shock	sPESI ≥ 1	Right heart strain on imaging	Cardiac biomarkers
**High** > **15%**		+	(+)	+	+
**Intermediate 3–15%**	**high**	−	+	+	+
	**low**	−	+	0-1 positive	
**Low ≤ 1%**		−	−	−	optional

Predicts 30-day outcomes of patients with PE. 1 point is given for age > 80, history of cancer, history of chronic cardiopulmonary disease, a heart rate ≥ 110, systolic blood pressure < 100 mmHg and O_2_ saturation < 90%

*sPESI* simplified pulmonary embolism severity index

## Treatment recommendations according to current guidelines

Clinical management of PE with high mortality risk includes cautious volume administration, aggressive oxygenation and treatment of hypercapnia, with a low threshold for vasopressors and inhaled pulmonary vasodilators for refractory hypoxemia. Intubation should be avoided due to the risk of transient hypoxemia, hypotension, sedation-related suppression of endogenous sympathetic activation and the risk of worsening right ventricular afterload from positive pressure ventilation [13, 14]. All patients should receive anticoagulation with unfractionated heparin. Based on robust meta-analyses registries, the 2019 ESC guidelines recommend systemic thrombolysis for high-risk patients or those of lower risk but signs of clinical deterioration (class 1b recommendation), while catheter-based procedures are reserved for cases where thrombolysis is contraindicated or ineffective (class IIa) [9, 15–17] (Fig. 1). Due to the high procedural success rates of novel thrombectomy devices and signals of reduced mortality and bleeding risks, a 2022 ESC consensus statement proposes interventional strategies as viable alternative to thrombolysis for deteriorating intermediate-risk patients, even in the absence of thrombolytic contraindications [18, 19]. Surgical embolectomy is reserved for cases with significant clot-in-transit or failure of less invasive alternatives with 30-day mortality and 5-year survival rates similar to thrombolysis in elected patients and experienced centers [20, 21]. The placement of cava filters for the prevention of further embolization belonged to the earliest interventions offered in PE and is still considered when anticoagulation is contraindicated or fails [9]. Veno-arterial extracorporeal membrane oxygenation (VA-ECMO) can serve as a bridging strategy for catastrophic PE until definitive treatment.

## Increasing role of pulmonary embolism response teams (PERTs)

The 2022 ESC consensus recommended the establishment of Pulmonary Embolism Response Teams (PERTs) for defining treatment goals due to a growing body of evidence suggesting PERT activation to improve short- and mid-term mortality as well as readmission rates [22, 23]. These interdisciplinary teams of experts in cardiology, pulmonology, hematology, vascular medicine, intensive care, cardiothoracic surgery or interventional radiology are meant to offer specialized care for patients with elevated mortality risk and to provide centralized, spoke-like referral structures for regional healthcare providers. At a minimum, a PERT requires an interdisciplinary intensive care department, ideally experienced with extracorporeal life support systems, a 24/7 on-site radiology department, and interventionalists with 24/7 availability at least on an on-call basis. Starting with one intensive care unit, one interventional department, and one catheter-based procedure, respectively, helps to avoid structural redundancies and management inconsistencies. Once a center has developed a standardized protocol—from PERT activation, case discussion, feedback-driven individual case management to follow-up—additional local treatment teams, as well as external referrals, can be seamlessly integrated into the system. FDA-cleared and CE-certified artificial intelligence solutions for automated image interpretation and clinical data integration can help streamline PERT activation and reduce the time-to-consult, but at the same time, still bear the risk of automation bias and notification fatigue if false positive rates are unsatisfactory [24, 25].

**Fig. 2:**
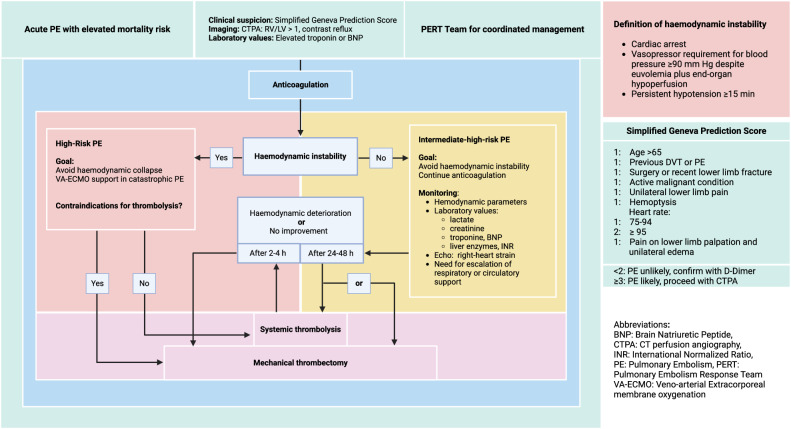
Treatment algorithm for elevated pulmonary embolism. Created in BioRender. Lüdemann, W. (2025) https://BioRender.com/p64u376

Besides risk stratification and procedural feasibility, part of a typical PERT discussion should be the individual patient’s disposition to tolerate an intervention lying supine and breathing spontaneously. Converting an intermediate-high-risk patient into a high-risk patient during a procedure or by means of prior elective intubation is a significant danger. Hemodynamically deteriorating patients should be promptly evaluated for reperfusion strategies by the PERT team before progressing to overt cardiogenic shock. Based on evidence of better outcomes for early compared to delayed interventions, a decision for thrombectomy should entail its start within 60–90 min or 2–4 h following systemic lysis [18, 26, 27] (Fig. 2).

## Intravenous thrombolysis: who is likely to benefit?

According to current guidelines, accelerated intravenous administration of alteplase (100 mg alteplase over the course of 2 h) is preferred to prolonged infusions of first-generation thrombolytic agents streptokinase and urokinase, which are less fibrin-specific and may require discontinuation of heparin administration [9, 28]. Systemic thrombolysis about halves the risk of cardiovascular collapse in high-risk patients, with a 1.5% risk of intracranial bleeding compared to an estimated 0.7% with anticoagulation alone [4, 15, 29, 30]. The risk is highest in patients with prior cerebrovascular events, cardiovascular diseases or advanced age [31]. The benefit of thrombolysis for intermediate-risk patients is unclear which is why this cohort is probably historically undertreated [32]. Although thrombolysis reduces short-term mortality and recurrent PE risk, no long-term benefit on mortality or cardiovascular outcomes, such as the incidence of pulmonary hypertension, has been demonstrated [30, 32]. Another aspect to consider when administering thrombolysis is that its effectiveness depends on the age and composition of the clot; older, connective tissue-rich thrombi are less susceptible to fibrinolysis, explaining failures of treatment response [32, 33]. The aforementioned 2020 registry study, which analyzed data from the German Federal Statistical Office between 2005 and 2015 including 885.806 patients, showed that thrombolysis reduced the risk of in-hospital death by 60% in unstable patients without prior resuscitation events, which is in line with historical data [4]. Although only about one-third of patients are expected to have contraindications to thrombolysis, it was ordered in only about 25% of patients post-resuscitation and 15% of unstable patients without prior resuscitation [4, 34]. The disproportionately rare use outside of experienced centers reflects a common dilemma and is probably owed to the challenges of risk stratification, fear of intracranial bleeding, uncertain individual efficacy and the lack of established “time is tissue” paradigms as in ischemic stroke or acute coronary syndrome. The recommended maximum doses for systemic thrombolysis in myocardial infarction, stroke and PE are similar, with the dosing calculation for PE being a transfer of experience from the other two vascular territories that receive only a fraction of the cardiac output [35]. Concepts using half or even a quarter of the standard dose were demonstrated to be safe and effective, particularly in patients with relative contraindications to thrombolysis and undergo further investigation, i.e., in the ongoing PEITHO-3 trial [29, 36–38].

## Catheter-directed thrombolysis

Local catheter-directed thrombolysis (CDT) aims to reduce bleeding risks while optimizing thrombolytic efficacy, using reduced doses typically infused over 24 h. The 2018 OPTALYSE study suggested doses as low as 8–12 mg alteplase may suffice [39]. The concept is to enhance thrombolytic efficacy through overcoming pulmonary shunting of systemically administered thrombolysis to regions with less thrombus burden. A randomized controlled trial (RCT) from the late 1980s found infusion of 50 mg alteplase directly into the pulmonary not superior to administration via a peripheral vein, likely due to shunting to the unobstructed contralateral artery [40, 41]. Interest in CDT sparked again with ultrasound-assisted CDT as in vitro studies suggested ultrasound to improve thrombolytic penetration into clots [42]. However, the recent SUNSET sPE trial demonstrated identical thrombus reduction with both standard and ultrasound-assisted CDT, questioning the clinical relevance of this mechanism [43]. A recent meta-analysis likewise failed to demonstrate an added value of ultrasound assistance to standard CDT [44]. Whether CDT without ultrasound assistance is superior to peripheral thrombolysis of the same dose remains unclear. A network metanalysis found CDT associated with reduced mortality and bleeding compared to systemic thrombolysis, though the only included RCT showed no statistical difference [45]. Another 2023 meta-analysis reached similar conclusions [46]. The problem is that most studies are retrospective or compare CDT with anticoagulation, limiting generalizability due to selection bias. Patients undergoing systemic thrombolysis are often more severely affected, and centers offering CDT usually have greater expertise in managing PE [47, 48]. It is possible that the first-pass effect of direct thrombolytic infusion into pulmonary thrombi has been overestimated and that the assumed benefits of CDT rather reflect the effects of low-dose systemic thrombolysis and second-pass re-circulation [49]. The most promising study to address this uncertainty is the ongoing Danish STRATIFY trial which is expected to conclude by the end of 2024 [50]. This study prospectively includes 210 intermediate-risk patients and compares ultrasound-assisted CDT, peripheral thrombolysis with a reduced dose of 20 mg rTPA, and anticoagulation alone. Other large-scale RCTs such as BETULA, PE-TRACT, and HI-PEITHO, are comparing different forms of CDT or CDT with anticoagulation with follow-up time frames of 12 months [51–53].

## Mechanical thrombectomy procedures

**Fig. 3:**
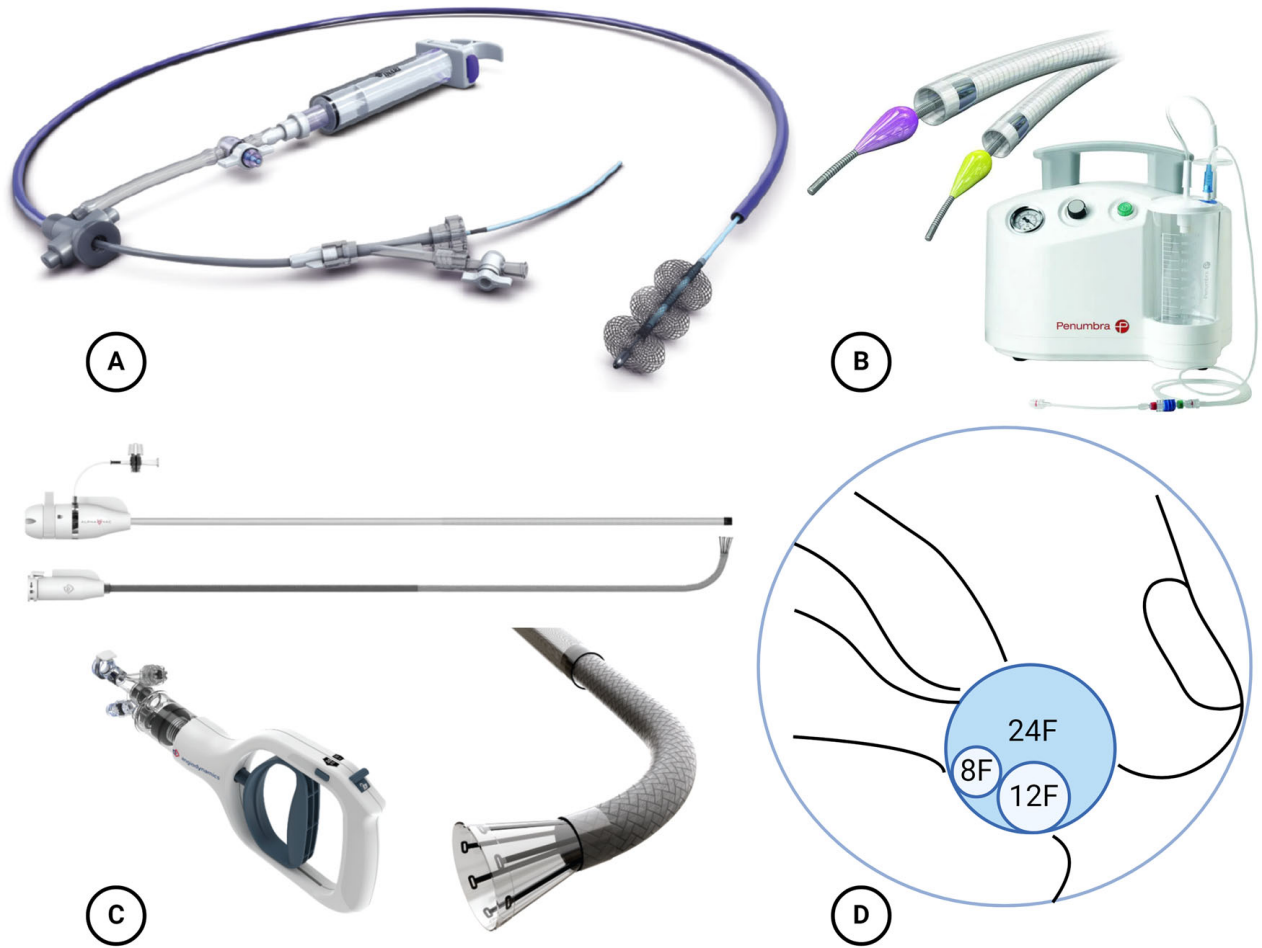
Modern devices for percutaneous thrombectomy. **A** The Inari FlowTriever system is a mechanical, large-bore aspiration catheter with diameters of 16F, 20F, and 24F. Sudden release of a 60 mL vacuum leads to aspiration of the thrombus into the connected syringe. A thrombus can be macerated or mobilized using self-expanding nitinol mesh discs prior to thrombectomy. **B** The Penumbra Lightning Indigo aspiration system is an electrically powered aspiration system with 6F, 8F, 12F and 16F (U.S.) catheter diameters. The continuous suction adapts automatically depending on the position in the thrombus or free vessel lumen in order to minimize blood loss. A so-called separator can be used coaxially to macerate thrombi. **C** The Angiodynamics AlphaVac System comes with a mechanical handle that works as the engine and offers 18F and 22F aspiration catheters. AlphaVac is already approved in the United States, with approval for Europe pending. **D** Illustration of different catheter sizes. The potential flow rate of a 24F system is eight times greater than that of a 12F system according to the Hagen–Poiseuille equation of fluid dynamics

In the 1990s, initial attempts at catheter-based fragmentation using rotating and hydrodynamic catheters emerged, followed by aspiration and ultrasound-guided local thrombolysis [26, 54]. A breakthrough in treating PE occurred with the introduction of novel aspiration catheters around 2019. The AngioVac system (AngioDynamics), a 24F aspiration catheter that reinfuses aspirated blood through an extracorporeal circuit, has been used for thrombus aspiration in PE but is only approved for the treatment of venous thrombosis. A refined follow-up device called AlphaVac is no longer dependent on an extracorporal circuit. It comes with a mechanical handle that works as the engine as well as with 18F and 22F aspiration catheters (Fig. 3). AlphaVac is already approved in the United States, with approval for Europe pending and currently being tested for pulmonary use in the APEX-AV study [55]. The two systems with the largest evidentiary basis to date, currently approved in both the United States and Europe, are the Inari FlowTriever System (Inari Medical) and the Lightning Indigo Aspiration System (Penumbra) (Fig. 3).

## Inari FlowTriever

**Fig. 4:**
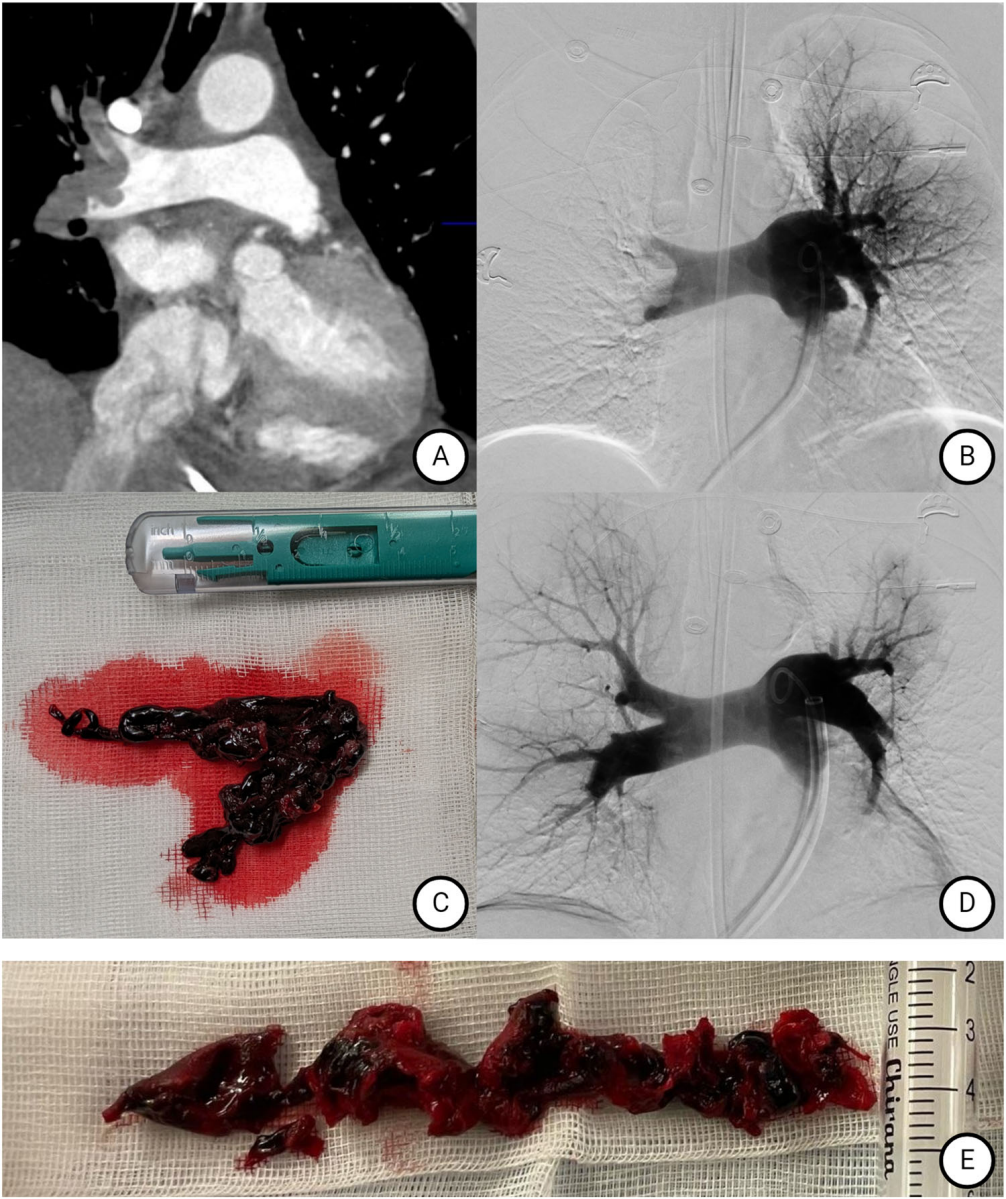
Large-bore thrombectomy in high-risk pulmonary embolism. **A** Pre-treatment CT-Scan. **B** Digital subtraction angiography (DSA) before thrombectomy. **C** Macroscopic thrombus after removal. Histologic analysis revealed it to be mostly fresh with about 20% fibrin content. **D** Final DSA after thrombus removal. **E** Thrombus from another patient who needed to be intervened due to failure of thrombolysis. Fibrin content was 70% (yellow portions of the specimen)

The FlowTriever system is a purely mechanical, large-bore aspiration catheter with diameters of 16F, 20F, and 24F. After placing a stiff guidewire into a lower lobe artery using standard angiographic techniques, the catheter is positioned in front of the clot, which can be macerated or mobilized using self-expanding nitinol mesh discs if aspiration alone fails. A 60 mL syringe is used to create a vacuum, its sudden release facilitates thrombus aspiration (Fig. 4). The aspirated blood can be manually reinfused through a filter. The system requires a learning curve for optimal results, as clots may get trapped, necessitating the removal of the whole catheter. The FlowTriever Pulmonary Embolectomy Clinical Study (FLARE), a prospective, single-arm, multicenter study of 106 intermediate-risk patients with signs of right heart strain, showed an average reduction in the right-to-left ventricular ratio (RV/LV ratio) of 25.1%, with a major adverse event rate of 3.8% [56]. The average intensive care unit (ICU) stay was 1.5 days. The 2023 FlowTriever All-Comer Registry for Patient Safety and Hemodynamics (FLASH) study included 800 patients in the US and 200 in Europe and confirmed these results [34]. In the US cohort, 92.1% were classified as intermediate-high risk, and 7.9% were high-risk, with 32.1% of patients having contraindications to thrombolysis. The peri-interventional RV/LV ratio decreased from 1.23 to 0.98 (*p* < 0.0001), and the mean pulmonary artery pressure (PAP) dropped from 48.9 mmHg to 38.8 mmHg (*p* < 0.0001). The median hospital stay was 3 days, with nearly two-thirds of patients not requiring ICU monitoring. Major adverse events at 48 h occurred in 1.8%, with 1.4% due to major bleeding before the introduction of the reinfusion filter system. The 30-day mortality rate was 0.8%, with no deaths attributed to the system or the procedure. The FLAME study, a prospective multicenter observational study in high-risk patients, showed fewer adverse events compared to cohorts with systemic lysis or anticoagulation alone [57]. The recently published PEERLESS study, an RCT of 550 patients with intermediate-high risk PE, compared mortality, adverse events, and ICU stays between CDT and aspiration thrombectomy. Aspiration thrombectomy had lower rates of clinical deterioration or bailout therapies as well as reduced intensive care unit admissions, hospital length of stay, and 30-day readmissions, with no difference in mortality or bleeding [58]. PEERLESS 2 and PERSEVERE are ongoing RCTs investigating thrombectomy versus anticoagulation in 1200 intermediate-high and 200 high-risk patients, respectively [59, 60].

## Penumbra lightning Indigo aspiration system

The Indigo aspiration system is an electrically powered aspiration system with 6F, 8F, 12F, and 16F catheter diameters. The continuous suction is modulated automatically depending on the position in the thrombus or free vessel lumen in order to minimize blood loss, whereas the BOLT technology, introduced in 2023 for the 7F system, adds pulsatile suction to improve clot disintegration. Given the smaller profile, handling and distal maneuvering are less challenging compared to the FlowTriever system. However, the system is not capable of removing complete thrombus through abrupt convective force, and smaller catheters might have a tendency of creating channels within the clot. Similar to the FLARE study results with the FlowTriever system, the EXTRACT-PE study, carried out with the 8F system in 119 intermediate-risk patients, showed a reduction in the RV/LV ratio by an average of 27 ± 13% (from 1.47 ± 0.30 before to 1.04 ± 0.16 afterward) [61]. The average reduction in systolic PAP was 4.3 mmHg (95% CI 2.6–5.9 mmHg, *p* < 0.0001), with an average procedure duration of 37 min (95% CI 23.5–60.0 min) and an ICU stay of 1 day. Severe bleeding events were reported in two patients, and pulmonary artery injuries occurred in two other patients, with an overall adverse event rate of 1.7%. The prospective STRIKE-PE observational study with the 12F system is ongoing to assess safety and effectiveness [62]. An interim analysis published in 2024 of 150 patients treated with the 12F system demonstrated a significant reduction in pulmonary artery pressure and RV/LV ratio, a median thrombectomy time of 33.5 min, a composite major adverse event rate of 2.7%, and significant improvements in 90-day functional and quality of life outcomes [63]. The first randomized study with the 12F system, the STORM-PE study, started recruiting at the end of 2023 and aims to enroll 100 intermediate-high-risk patients by the end of 2026, comparing thrombectomy with anticoagulation alone [64]. The primary endpoint is the RV/LV ratio after 48 h. Furthermore, the CATH-PE case-control study will examine 100 patients with high or intermediate-high risk [65].

## Conclusion and outlook

The pathophysiology of life-threatening PE is a spiral of an acute increase in right ventricular afterload, ballooning, and increased wall tension of the right ventricle, leading to reduced perfusion, septal shift, impaired left ventricular function, and thus systemic malperfusion, eventually causing obstructive shock with right heart failure. High-risk patients should be offered full-dose thrombolysis in the absence of contraindications. Intermediate-risk patients not improving or deteriorating on anticoagulation alone, as well as high-risk patients with at least relative contraindications to thrombolysis, should be evaluated for thrombectomy. To date, the available data is not compelling enough to recommend catheter-directed thrombolysis as the interventional strategy of choice. Bleeding risks are still elevated compared to anticoagulation alone, and the incremental benefit of CDT over reduced-dose thrombolysis via a peripheral vein, which can be performed in the safe environment of an ICU, is unclear. Modern thrombectomy techniques have proven effective in reducing right ventricular afterload and improving hemodynamic and functional parameters. The goal of interventional treatment, based on current data, is to reduce right ventricular afterload or achieve hemodynamic stabilization, not necessarily complete thrombus clearance from the pulmonary arteries. However, for novel thrombectomy procedures only non-interventional observational studies are available so far. While these results are impressive, RCTs are lacking for the routine recommendation of thrombectomy in high-risk patients, potentially as an alternative to thrombolysis. For the two systems with the largest evidentiary basis to date, the Inari FlowTriever and the Penumbra Indigo Aspiration Systems, these trials are underway (Table 2). High-risk patients remain the most challenging cohort to study since it is ethically difficult to perform RCTs in patients with life-threatening conditions as these studies are likely to suffer from high bias due to crossover between treatment arms. Acute PE not only carries significant mortality but also causes considerable morbidity due to post-PE syndromes and CTEPH. Whether modern catheter techniques can reduce the incidence of these conditions by decreasing thrombus burden and how this might influence the technical endpoints of intervention as well as patient selection is also the subject of ongoing studies.

**Table 2:** Ongoing randomized controlled trials of thrombectomy in pulmonary embolism

Study identifier (ClinicalTrials.gov)	Device	Design	Follow-up	Cohort	Primary outcome measures
PE-TRACT NCT05591118	MT (any) CDT (any)	MT (any)+AC vs. CDT (any)+AC vs. AC alone	12 months		Peak oxygen consumption (month 3) NYHA class (month 12) Major bleeding (up to day 7)
STORM-PE NCT05684796	Indigo Aspiration System	MT + AC vs. AC alone	90 days		Change in RV/LV ratio at 48 h on original therapy as assessed by computerized tomography pulmonary angiogram (CTPA)
PEERLESS NCT05111613	FlowTriever	MT + AC vs. CDT + AC	1 month		Composite clinical endpoint constructed as a win ratio (7 days): All-cause mortality, or Intracranial hemorrhage or Major bleeding or Clinical deterioration or ICU admission and ICU length-of-stay
PEERLESS 2 NCT06055920	FlowTriever	MT + AC vs. AC alone	3 months		Composite clinical endpoint constructed as a win ratio (30 days): Clinical deterioration All-cause hospital readmission Bailout therapy Dyspnea (48 h)
PERSEVERE NCT06588634	FlowTriever	MT + AC vs. Standard of care	3 months		Composite clinical endpoint of the following adjudicated events (7 days): All-cause mortality Cardiac arrest Bailout to an alternative therapeutic strategy Major bleeding ECMO life support

In orange: intermediate-high-risk cohorts, in red: high-risk cohort

*AC* anticoagulation, *CDT* catheter-directed thrombolysis, *CTPA* CT pulmonary angiography, *ECMO* extracorporeal membrane oxygenation, *ICU* intensive care unit, *MT* mechanical thrombectomy, *NYHA* New York Heart Association, *PE* pulmonary embolism, *RV/LV* right-to-left ventricular

## Abbreviations

CDT: Catheter-directed thrombolysis
CTEPH: Chronic thromboembolic pulmonary hypertension
ESC: European Society of Cardiology
ICU: Intensive care unit
PAP: Pulmonary artery pressure
PE: Pulmonary embolism
PERT: Pulmonary embolism response team
RCT: Randomized controlled trial
sPESI: Simplified pulmonary embolism severity index
