# Correction: Acute pulmonary embolism: a paradigm shift in interventional treatment and interdisciplinary care?

**DOI:** 10.1007/s00330-025-11805-9

**Published:** 2025-09-25

**Authors:** Willie M. Lüdemann, Federico Collettini, Uli Fehrenbach, Timo A. Auer, Maximilian de Bucourt, Bernhard Gebauer

**Affiliations:** Department of Diagnostic and Interventional Radiology, Charité Universitätsmedizin Berlin, corporate member of Freie Universität Berlin and Humboldt-Universität zu Berlin, Augustenburger Platz 1, Berlin, Germany

**Correction to: European Radiology**

10.1007/s00330-025-11548-7, published online 09 May 2025

In this article the author’s name Willie M. Lüdemann was incorrectly written as Willie M. Luedemann. Furthermore, in this article, author Timo Alexander Auer’s middle name was not given by mistake. The original article has been updated.

